# EU geographical islands as leaders of green energy transition

**DOI:** 10.12688/openreseurope.18856.2

**Published:** 2025-07-04

**Authors:** Giorgio Bonvicini, Fabiola Roccatagliata, Mario Cortese, Kostas Karanasios, Panos Kotsampopoulos, Fausto Sainz, Nora Ganzinelli, Alessandra Montanelli, Francesca Battistelli, Cristina Barbero, Emilio Ghiani, Sara Ruffini, Alessandra Cuneo

**Affiliations:** 1 RINA Consulting S.p.A., Genova, Italy; 2 R2M Solution S.r.l., Pavia, Italy; 3 DAFNI, Athens, Greece; 4 National Technical University of Athens, Zografou, Greece; 5 COMET Global Innovation S.L., Barcelona, Spain; 6 SINLOC S.p.A., Padova, Italy; 7 Institute of Atmospheric Pollution Research, Italian National Research Council, Montelibretti, Italy; 8 Comune di Berchidda, Berchidda, Italy; 9 Universita degli Studi di Cagliari, Cagliari, Italy

**Keywords:** Islands, energy transition, decarbonization, energy communities, energy storage, renewables, energy efficiency

## Abstract

This paper reviews how European islands are taking the lead in the European Union (EU) Clean Energy Transition by reviewing the lessons learned in the EU Bridge initiative and in a number of EU co-funded projects such as NESOI, RE-EMPOWERED, REACT, IANOS, LOCALRES, MASTERPIECE, SINNOGENES, SMHYLES, STEPWISE, and ISLET. Islands encounter significant difficulties in the management of their energy systems, including strong seasonal variations in energy demand, high operational costs and GHG emissions for energy production, weak energy grids, lack of technical skills, and difficult access to finance. However, they also have positive features that make them ideal laboratories for energy transition, including high potential for renewables, small-scale and strong community structures, and high energy prices, which make most solutions cost-effective. Each of the projects contributing to the paper has been supporting the islands’ energy transition, leveraging different enabling technologies, such as renewable energy production systems, smart grids, advanced energy storage systems, and local energy community schemes. The results from these projects underline the need for tailored energy planning, considering geographical and socio-economic particularities, the need to engage the local population in the definition of the most suitable decarbonization pathways for the island, and a number of lessons learned on the technologies that have the highest potential for being tested on islands and then being replicated on the mainland. Therefore, this study concludes that renewable energy solutions coupled with different technologies (storage, mobility, district heating/cooling, etc.) and leveraging powerful community integration confirm that European islands can drive the decarbonization strategy of the EU.

## Plain language summary

This paper draws lessons learnt from a set of various EU-funded projects, underlining how European islands are now playing a leading role in the transition to clean energy. These islands face unique challenges with regards to managing their energy systems, including fluctuating energy demand due to tourism, high energy production costs and related emissions, weak electricity grids, limited technical expertise and difficult access to finance. However, the advantages that they have-abundant renewable energy potential, tight-knit communities and high energy prices make them ideal playgrounds for the testing of innovative energy solutions.

The projects presented in the study have supported the adoption on islands of renewable energy systems, smart grids, advanced energy storage, and community-based energy enterprises. Key recommendations stress the need to tailor energy plans according to each island's geographical and social particularity and to actively involve residents in shaping decarbonization strategies.

The findings suggest that, together with the technologies of energy storage, electric mobility, and district heating or cooling, renewable energy can turn islands into powerful drivers of EU decarbonization. Islands are pioneering solutions that work for their specific needs but at the same time test ideas that might be scaled up and applied across the mainland.

## Disclaimer

The views expressed in this article are those of the authors. Publication in Open Research Europe does not imply endorsement of the European Commission.

## Nomenclature

**Table:** 

API	Application Programming Interface
BTM	Behind-the-Meter
CEN	European Committee for Standardization
CRM	Critical Raw Materials
CWA	CEN Workshop Agreement
EU	European Union
EV	Electric Vehicle
FTM	Front-the-Meter
GHG	GreenHouse Gas
HESS	Hybrid Energy Storage System
IPCC	Intergovernmental Panel on Climate Change
IRENA	International Renewable Energy Association
KNX	Konnex
LEC	Local Energy Community
LH	LightHouse
NESOI	New Energy Solutions Optimised for Islands
NGO	Non-Governmental Organisation
P2G	Power-to-Gas
R&D	Research and Development
REC	Renewable Energy Community
RED II	Renewable Energy Directive II
RES	Renewable Energy Sources
RFB	Redox Flow Battery
RRL	Replication Readiness Level
V2G	Vehicle-to-Grid
VRFB	Vanadium Redox Flow Battery

## Introduction

Since the creation of the Intergovernmental Panel on Climate Change (IPCC) in 1988, the global community has undertaken the challenge of limiting greenhouse gas (GHG) emissions into the atmosphere to reduce the effect of climate change, respecting the globally established objectives of the Paris Agreement to limit the rise of the global temperature to a maximum of 1.5°C above pre-industrial levels.

In this context, the European Commission is taking actions to make Europe the first climate-neutral continent by 2050, and among other actions to achieve this ambitious target is to make islands the locomotives of the European Union (EU) energy transition. According to recent data from the European Parliament, the total population of island regions in the EU territory is around 20.5 million people, of which 15 million are considered to live in small energy markets ^1^. Annex 2 of the Clean Energy for All Europeans Package makes special reference to the potential of islands to contribute to Europe’s energy transition ^2^. The European islands are in a unique position as early adopters of the European Green Deal and catalysts for renewable energy transformation ^3^. Non-interconnected small islands are strongly dependent on diesel and other oil products to satisfy their energy requirements. Fuels are imported by boat on islands, and the small-scale energy production and logistics issues of fuel supply on islands result in very high comparative energy costs ^4^.

Guaranteeing a stable energy supply, independent of fossil fuels, in contexts such as small/medium-sized islands, remains a difficult task. The geographical separation of their power systems from the mainland energy markets, combined with the scarcity of local technology and skills, is the main problem to tackle in the years to come ^5^.

Despite this, small islands are emerging as the best lab-testing territories on which renewable sources can help overcome this problem, offering suitable clean energy solutions and policy tools for reduced power markets to be scaled efficiently in other islands with similar features or even across mainland regions. This should stimulate financial mechanisms that will enhance the adoption of advanced renewable solutions and reduce the dependence on high-carbon-emitting sources.

The European Commission is funding many projects aimed at promoting the decarbonization of island energy production networks and collaborating with international organizations such as the International Renewable Energy Agency (IRENA) ^6^. The novelty of the present paper is the collection of lessons learnt from a large number of EU co-funded research and development projects on energy transition of islands to support the achievement of the islands’ decarbonization targets.

## Challenges and opportunities for Islands’ energy transition

Isolated regions such as small islands are acknowledged to be highly exposed to climate change risks, falling behind in the energy transition due to the slow implementation of fossil fuel alternative technologies in energy generation, in comparison with mainland regions, and the limited availability of space to develop them. The complexity of the energy demand in these regions is characterized by a heterogeneous distribution throughout the year, with high peaks due to tourism, creating a strong reliance on non-renewable energy sources conveyed by sea carriers, resulting in an increase in GHG emissions and high freight charges per unit produced ^7, 8^. In addition, the difficulties in public administration, whose authorities are often separated from the mentioned regions, and the lack of expertise regarding energy transition-specific planning results in a deceleration on the path to decarbonization and limiting energy self-sufficiency.

Islands have generally been identified as territories with significant potential for renewable energy sources (e.g. due to the higher solar radiation in the Mediterranean region, the high wind potential in northern Europe, etc.). The high costs of carbon-intensive solutions compared with inland regions make it more feasible to study the implementation of sustainable energy generation systems, which are easily managed because of smart grid solutions applicable to separate power markets ^9^. Moreover, these populations are characterized by strong community bonds, facilitating social cohesion to build a resilient and autonomous energy network ^10^.

In this framework, the knowledge of tested energy transition solutions and methodologies developed by European initiatives, added to the crescent amount of private and public investment strategies, such as the European Structural Investment Funds or the European Funds for Strategic Investments, should enhance the potential of the island to become one of the main drivers of the EU in sustainable development and clean energy research laboratories.

## EU projects experience on Islands’ energy transition

The following paragraphs present the main outcomes of several EU co-funded initiatives and projects that have worked or are working on the energy transition of islands. For each project, a brief introduction, details of the demonstrated technologies, and the main lessons learned were provided.

Table 1 presents a comparison of the main features of the analyzed projects on energy transition of islands.

**Table 1.** Comparison of the Analyzed Projects.

Project/Initiative	Technology	Islands’ Geography
BRIDGE	smart grids, energy storage, digitalization, local energy systems	EU-level
NESOI	energy planning, renewable energy production, energy storage, energy communities, sustainable mobility, green hydrogen	54 projects in 70 different EU islands
RE-EMPOWERED	multi-microgrid applications	two demo sites in Europe (Denmark, Greece) and two demo sites in India
REACT	local energy communities	two demo sites, in Italy and Spain
IANOS	renewable energy production, energy storage, grid flexibility services	two demo sites, in the Netherlands and Portugal, and three replication sites in Italy, Greece and France (overseas territories)
STEPWISE	clean energy transition plans	three demo sites, in Croatia, Greece and Malta
ISLET	renewable energy communities	three demo sites in Italy, Croatia and Greece
MASTERPIECE	local energy communities	four demo sites, in Sweden, Turkey, France and Italy
LOCALRES	renewable energy communities	two demo sites, in Netherlands and Italy
SINNOGENES	energy storage	two demo sites, in Greece
SMHYLES	energy storage	demo site in Portugal

### BRIDGE

The BRIDGE initiative, facilitated by the European Commission, is a collaborative platform joining research and innovation projects across Europe in the fields of smart grids, energy storage, digitalization, and local energy systems. This initiative was designed to leverage the combined insights and technical advancements of these projects to accelerate Europe’s clean energy transition. By fostering coordination, BRIDGE enables projects to pool knowledge, address common regulatory and technical barriers, and develop best practices that can be adopted widely to create flexible, secure, and affordable energy systems.

Islands play a central role in the BRIDGE initiative due to their unique status as "energy islands," a term describing regions that are either fully autonomous or possess limited connectivity to larger energy grids. These locations face unique challenges such as dependency on imported fuel or high vulnerability to supply interruptions, which make them ideal candidates for innovative energy solutions focused on resilience and sustainability. Through BRIDGE, islands can participate in dedicated multi-energy planning projects that deploy diverse cross-sector energy solutions. These projects aim to build locally integrated energy systems that combine renewables, storage, and digital management tools to reduce carbon dependency, while securing a reliable energy supply for island communities.

In practice, these island-focused projects within BRIDGE are piloting a range of new solutions, including systems that can effectively combine multiple energy vectors (such as electricity, heat, and hydrogen), enhance local renewable generation, and manage demand through digital tools. The projects were structured to validate these technologies under real-world conditions, allowing islands to serve as testbeds for innovations in decarbonized and decentralized energy management.

The lessons learned from these pilot projects have been proven invaluable. First, they underline the advantages of integrated, multi-vector energy systems that maximize resource efficiency by flexibly shifting between energy forms, depending on demand and supply. Furthermore, the projects highlight the importance of community engagement, involving local residents not only in improving the acceptance of new technologies but also in strengthening local ownership and participation in the energy transition. Finally, these island initiatives reveal the critical need for adaptable policies that can accommodate both the technical specifics of new energy solutions and the local characteristics of island communities, such as environmental constraints and the high cost of infrastructure expansion.

By capturing and sharing these insights, BRIDGE built a repository of best practices, providing a foundation for the replication and scaling of these solutions in other isolated or grid-constrained areas across Europe. These projects not only enhance energy security and sustainability but also set the stage for future advancements. The resulting solutions not only enhance resilience and sustainability in isolated systems, but also contribute to Europe’s overarching climate goals, supporting a just and community-driven transition to renewable energy across diverse geographies.

### NESOI

The EU Islands Facility (New Energy Solutions Optimised for Islands – NESOI) aims to facilitate the decentralization of energy systems on EU islands and contribute to 2030 climate targets by mobilizing over 100 million € of investment in sustainable energy projects. NESOI organized two open calls for beneficiary projects, in 2020 and 2022, whereby 166 island energy-transition project proposals were evaluated, and 54 beneficiary projects were selected by NESOI experts according to transparent and rigorous criteria. These projects span 12 European countries and cover a wide range of energy-transition topics, innovative technology deployments, and diverse maturity levels. The 54 supported projects were located over a total of 70 islands, and the technical and financial assistance provided helped mobilize investments for 455 million €, out of which 88 million € were already identified.

Finally, NESOI has delivered a digital platform designed to stimulate collaboration, open innovation, and visibility of investment opportunities. It includes a suite of scalable components, such as profile and smart matching, online collaboration space and capacity building, e-learning, and equity crowdfunding. It is the most visible and important tool of the facility as it helps local operators develop their own projects.

The NESOI image and its approach have become well-known ecosystems among the EU geographical islands. The extensive visibility gained over time can ensure the opportunity for the facility to promote some immediate outputs and continue the effort to build a standard framework.

To leverage these results, the first ongoing activity after the completion of the project is related to the inclusion of NESOI within the European Standardization System, through a CEN Workshop Agreement on the “Standardized Approach for the Management Optimization of a Technical Assistance Facility.” To this end, three key Italian partners from the NESOI Consortium (R2M Solution, RINA, SINLOC) put together forces to develop a CEN Workshop Agreement (CWA).

A CWA is the process by which CEN publishes and formalizes a reference practice within a specific domain; it is advantageous for addressing dynamic and rapidly evolving fields within the European market because it allows us to address industry needs rapidly, fostering innovation and adaptation to new methodologies, processes, and technologies.

Among other key topics, the CWA will include the NESOI model to assess the replicability of supported island projects. This approach predicts that projects will be clustered into five main areas:

- energy planning;
- renewable energy production and energy storage;
- energy communities;
- sustainable mobility;
- hydrogen.

The approach also introduces the Replicability Readiness Level (RRL), which is an indicator that simplifies the assessment by assigning a score to each replication area:

- geographical;
- technological;
- legal;
- social;
- economic/financial.

The detailed process and selected best practices are available in the NESOI Guidebook for Replication of Islands’ Energy Transition Projects.

### RE-EMPOWERED

The RE-EMPOWERED project aims to develop and demonstrate novel tools to provide a complete solution for all stages of microgrid/energy island and multi-microgrid applications. The tools include energy planning, ranging from the design of microgrids from scratch to the upgrade of existing installation to high-renewable energy sources (RES) systems. The tools and solutions are demonstrated in four demo sites with weak or absent grids, two in Europe (Bornholm in Denmark and Kythnos in Greece), and two in India (Keonjhar and Ghoramara).

In the frame of the project, a microgrid in Ghoramara demo site was created mainly consisting of PVs (150 kW), with the inclusion of storage (720 kWh) and small wind turbines (10 kW). 

The Keonjhar microgrid was also renovated with the inclusion of new PVs (30 kW), battery storage (180 kWh), a biogas station (10 kW) and a biomass station (10 kW).

The innovative solutions developed in the project include:

- ecoEMS: Improving the efficiency of weakly interconnected energy systems by integrating renewable energy sources (RES) and storage;
- ecoMicrogrid: Optimization of microgrids through advanced algorithms for managing RES, loads, and storage;
- ecoPlanning: A decision-support tool for mid-term energy planning in Non-Interconnected Islands, assessing new conventional and RES integration and interconnection benefits;
- ecoDR: A smart metering and load control system enabling dynamic pricing, demand-side management, and remote load control in residential settings;
- EcoPlatform: A cloud-based platform providing secure management of energy infrastructure, integrating RE-EMPOWERED tools, and handling data streams;
- ecoMonitor: A portable, sensor-equipped platform for real-time air and water quality monitoring, integrating with ecoTools for analysis and supporting solar-powered water purification in Indian demo sites;
- ecoCommunity: A digital platform fostering citizen engagement and participation featuring dynamic pricing, load management, billing, and community feedback mechanisms;
- ecoresilience: Focuses on developing cyclone-resilient structures for solar PV and wind systems using local resources for improved maintenance and resilience;
- ecoConverter: Develop power converters for microgrids, optimize energy extraction from PV systems under partial shading, and provide ancillary grid services;
- ecoVehicle: Establish charging stations and deploy electric vehicles to support green transportation on Ghoramara Island and nearby areas.

The main lessons learnt during the project include:

- It is well established that off-grid systems often fail in the long run because of social rather than technical issues;
- In the project implementation, it was very clear that engaging the local communities in the activities as early as possible was a crucial element for the project’s success. This was particularly important for the off-grid Indian demo-sites, where additional challenges were present, such as limited energy literacy and different local languages;
- It was understood that comprehensive planning is important, including the transfer of knowledge to the local community and financial sustainability;
- a community awareness program was carried out to inform the local citizens on the efficient use of the system, while tailor-made training to the pilot-site operators was performed; the operator training involved training on both the operation of the basic infrastructure (inverters, storage etc), but also on the advanced ecoTools developed in the project;
- European and Indian cooperation was a great opportunity for mutual learning and growth, which was achieved by recognizing the complementarity of expertise of the India and EU research groups and combining it for the success of the project
- Finally, emphasis was placed on the versatility and flexibility of the features of ecoTools in order to maximize their compatibility and replicability in similar locations.

### REACT

The aim of the REACT project was to support island communities in achieving energy independence by developing a technical and business model to demonstrate that the large-scale deployment of RES and storage assets, coupled with an ICT platform to enable an integrated and digitalized smart grid, can bring economic and environmental benefits to their local energy communities. Hardware and software were installed in the buildings of all participants and monitored throughout the project. It is important to underline the importance of understanding participants’ starting points in terms of awareness and knowledge when planning and implementing energy transition activities ^11^. Interviews were conducted to determine the participants’ knowledge and ideas on energy-related management, energy storage, energy savings, and general knowledge about energy. They all perceived themselves as being informed about energy savings, but less so about energy management and storage. All were concerned about energy prices, especially in Inis Mor, where they had a strong sense of community. In San Pietro, environmental protection and savings (both economic and energy-related) were mentioned as positive aspects of the proposed solutions. In La Graciosa, most participants were initially reluctant to participate and were then very satisfied with the energy independence results ^12^. Innovative business models and exploitation plans have been developed and deployed to increase the penetration of RES, reduce fossil fuel consumption, allow for large-scale replication, and enhance autonomy for islands while contributing to Europe’s energy security, paving the way for regulatory and legal challenges.

REACT engaged and involved island residents in demand reduction and time-shifting activities that raised their awareness and allowed them to become an active part of the cooperative strategy.

The main lessons learnt during the project include:

- involve all stakeholders in informative meetings, as REACT did in the three pilots, so that they can explain things in simple language to the general population.
- it is important to speed up permissions at local, regional and national level;
- Provide very clear information to prospective users of the system or to those interested in green installations, whether solar, hydrogen, geothermal, or any other green energy solutions;
- Make sure in small places such as small remote island maintenance and repairs are available at reasonable times and prices;
- Trust is a very important factor in engaging people in activities, enabling participation and engagement.
- energy communities can also strengthen the local community.

The implementation of the REACT solution was successful for all participants and opened the door for creating energy communities on small islands, as well as for scaling up solutions for larger islands.

### IANOS

The IANOS project was designed to tackle the unique challenges faced by island environments through a range of technological and non-technological solutions. By testing these solutions in real-world conditions, the project aims to address various climatic and socioeconomic factors, focusing on diverse energy supply, storage, and end-use vectors. Demonstrations are set to take place on two Lighthouse Islands: Ameland in the Netherlands and Terceira in Portugal, with a replication strategy targeting Bora-Bora, Lampedusa, and Nisyros.

The heart of IANOS is an intelligent Virtual Power Plant that coordinates multiple energy resources, including RES, smart grid solutions, and multi-vector energy storage. This approach enhances self-consumption and flexibility for users, while promoting a Local Energy Community (LEC) framework that fosters collaboration and knowledge sharing. The project implemented nine Use Cases across the Lighthouse Islands, organized into three Transition Tracks:

- energy efficiency and grid support: managing high renewable energy penetration;
- Decarbonization: Focusing on electrification and non-emitting fuels for transportation, industrial loads, and heating networks.
- empowering local energy communities: encouraging citizen engagement in the decarbonization process.

The IANOS project has made notable progress in capacity building through a series of training sessions conducted on both the Lighthouse (LH) and Fellow islands. These sessions covered a mix of technical and non-technical topics, with sessions hosted on each LH island focusing on best practices, and on each fellow island showcasing initiatives from the LH islands. The primary goal was to support decarbonization efforts by drawing on lessons learned from the LH islands and equipping local stakeholders with the skills to engage citizens in sustainable energy decision making. To ensure maximum effectiveness, best practices from successful initiatives on the LH Islands were adapted to fit local contexts. The process began with a thorough assessment of each island's objectives and stakeholders’ needs. This was followed by stakeholder mapping to understand the roles and requirements of the various participants in energy sustainability. The participants were carefully chosen to facilitate effective engagement. One key influence on the project’s approach was the Samsø community energy initiative, which highlighted the importance of involving local residents in planning and decision-making ^13, 14^. IANOS adopted several successful strategies from Samsø, such as engaging local leaders to build trust, maintaining open communication through community sessions, allowing flexibility in planning to accommodate community feedback, and providing co-ownership opportunities in renewable energy projects to ensure local investment and benefits. The workshops focused on engaging different groups, including citizens, stakeholders, and schools. Efforts to raise awareness among citizens emphasize climate change, renewable energy, and sustainable practices. Connections were made between citizens and decision makers to encourage participation in energy innovation. Schools were also a key focus, with training sessions designed to inspire students and staff to take action in communities and homes. Before the sessions began, island representatives visited Samsø to learn about effective community engagement strategies. In a "train-the-trainer" approach, island partners received best practices shared by the Samsø Energy Academy and its founders. For islands such as Ameland, which already had advanced community engagement processes, these sessions provided crucial capacity-building support. Despite some challenges in recruitment, the training sessions successfully informed and engaged citizens using digital platforms, social media, and community events with food to create a welcoming environment ^15^. Presentations were kept brief to encourage discussion, and topics were made relatable by linking climate and energy issues to local experiences such as extreme weather events and rising energy costs. Stakeholder engagement involves formal and informal community leaders recruited through local municipalities and IANOS contacts. These sessions featured presentations followed by interactive discussions to encourage collaboration between decision makers, local associations, non-governmental organisations (NGOs), and businesses. In schools, energy and climate topics are made enjoyable through games and competitions, with teachers involved in planning to ensure impact. Students were encouraged to act as ambassadors for sustainable change with incentives such as t-shirts or certificates to reinforce their learning.

The IANOS project yielded valuable insights through its training initiatives, leading to several key takeaways.

- Engagement is crucial: involving local residents in decision-making processes builds trust and encourages participation;
- tailored approaches matter; best practices must be adapted to local contexts for maximum effectiveness;
- effective communication: open dialogue fosters community ownership and enhances understanding of energy initiatives;
- Flexibility in planning: Adaptability to community input ensures that projects remain relevant and impactful;
- Co-ownership opportunities: enabling locals to invest in renewable energy enhances community buy-in and addresses broader needs;
- Diverse recruitment strategies that utilize various recruitment channels, including social media and local events, can overcome challenges in participant engagement;
- Relatable content: Making climate issues relatable to local experiences increases participant interest and engagement;
- Empowerment through education: Training students as sustainability ambassadors encourages behavioral changes within families and communities.

### STEPWISE

StepWise is a tailored and dynamic capacity-building program that transforms local and regional authorities into autonomous early adopters of digitized, integrated, and ambitious Clean Energy Transition Plans. The project was funded by the LIFE program and was launched in December 2023.

The aim of the project is to promote energy transition by building and increasing the skills and capacities of local and regional authorities to deliver, implement, and monitor their energy plans in their local context. StepWise intends to implement a use-case-based approach to develop a digital toolkit which consists of a “non-technical component,” including the knowledge repository and the training materials, and a “technical component,” which is a digital tool. The tool fits the typical stages of plan development, particularly:

- Stage 1 – Create a baseline model: the toolkit helps in pre-filling data that are difficult to retrieve to create the baseline scenario;
- Stage 2 – Create scenario intervention model(s): The toolkit enables analysis of the baseline scenario and allows the creation of intervention scenarios for various years;
- Stage 3 – Visualize carbon- and energy-related metrics: The toolkit accurately simulates the impact of the interventions on energy and carbon over the years. It also enables visualization of key results;
- Stage 4 – Visualize the map: The toolkit enables visualization of key energy and carbon metrics as a roadmap.

The project addresses municipalities in four use cases: Bulgaria, Spain, Cyprus, and the Mediterranean islands.

SINLOC is leading the use case on the Mediterranean islands. Currently, the use case involves three pilots: Cres, Crete, and Malta. As well represented by other initiatives, islands share some common features that characterize the use case in terms of the energy market, energy needs, size, population, governance, infrastructure, and capabilities available in the public and private markets, attaining a clean energy transition. Among these:

- willingness to promote energy self-reliance of islands & reduce dependency on fossil fuel imports;
- strong seasonality in the consumptions;
- needs to attract finance (project fragmentation, lack of tailored funds, higher costs, etc.).
- need for short-, medium-, and long-term planning (coordination between local authorities and projects, limited availability of areas, etc.).
- absence of technical, procedural, financial skills and competencies;
- governance instability.

Given these peculiarities, capacity building on energy planning becomes even more important and StepWise has the ambition to support local and regional authorities in gaining knowledge and develop their own energy plans.

The StepWise approach relies on a two-waves capacity building program: first local stakeholders (replicators) will be selected and trained to the StepWise toolkit, second municipalities (adopters) will be trained by the replicators and some of them will have their plan developed by the toolkit.

In this phase, the StepWise project is identifying local replicators, i.e. Energy Agencies and National Authorities, that will gain:

- Active involvement in all project phases to influence the development of the programme and toolkit and obtain early access to the Digital Toolkit through demos and workshops;
- Knowledge sharing and visibility by participating to workshops presenting project progress and live demonstrations of the toolkit and having the opportunity to have their logo and link on the Project website to highlight their commitment to energy transition;
- Research-backed capacity building and expertise and participation to a Steering Group to collaborate directly with trainers and technology providers;
- Training and leadership support for crafting effective Clean Energy Transition Plans (CETPs).

### ISLET

The ISLET project moves from the idea that Renewable Energy Communities (RECs), as defined in Directive 2018/2001 on the promotion of the use of energy from renewable sources (RED II) ^16^, can help small islands to overcome typical barriers for such territories; indeed, many islands suffer issues such as social abandonment and a lack of islanders’ high skills, the absence of energy market options, as in non-interconnected islands and some institutional isolation from the mainland ^17^.

At the same time, the perceptions that island communities hold towards renewable energy sources and intersect with specific imaginaries and identities of "islanders" ^18^. The engagement of communities in energy projects should consider the specific barriers and features of small islands, which is the goal of the ISLET project. The presence of key stakeholders and leaders can enhance the success of projects, and the islands’ municipalities can play a crucial role in implementing Renewable Energy Communities together with or beside citizens.

Thus, ISLET aims to support collaboration between public authorities, private investors, and citizens to develop RECs on the small Mediterranean islands.

More concretely, the project aims at:

- Training at least 70 representatives and staff of at least 30 island local authorities;
- creating seven RECs in three pilots (Procida, Cres, and Astypalea) and four test islands (in Italy, Greece, Croatia, and Malta), involving approximately 180 householders;
- providing Guidelines and policy recommendations for RECs in small islands at EU level;
- setting up five help desks at the EU level to support local authorities and citizens of the mall islands in the development of RECs;
- assessing the local impact RECs in pilot islands.

The ISLET project will define the model for Mediterranean small islands and spread it to all EU islands and key stakeholders to exploit the experience in as many islands as possible.

So far, the main lessons learned are related to RECs projects typical for small Mediterranean islands, although a one-size-fits-all solution does not exist due to different legal frameworks suffering from different problems:

- islanders look more at other small islands’ experiences than at closer experiences on the mainland.
- Small islands could be a perfect laboratory for RECs for any experience of social innovation.
- In a small island, the water-energy nexus addressed at the same time in the design of energy community could enhance the efficiency and the impact of a RECs; For RECs in small islands in the Mediterranean basin, the involvement of municipalities plays an interesting role, as it could provide school roofs close in summer when the islands generally reach the energy demand peak.

### MASTERPIECE

The Masterpiece project aims to create a digital coordination and cooperation modular platform of services that will facilitate the creation and operation of energy communities. The platform focuses on five pillars: innovation, user-centric solutions, cyber-security infrastructure, applicability, replicability, and business opportunities.

The project has four strategic pilot projects based in Sweden, Turkey, France, and Italy. In Italy, the pilot is particularly interesting: Berchidda, a small municipality in northern Sardinia, fully owns its electricity grid and has installed new smart meters replacing old ones, all of which went under renovation and are now completely remotely controlled, positioning Berchidda in a prominent position for the implementation and use of the Masterpiece platform.

**Figure 1.**
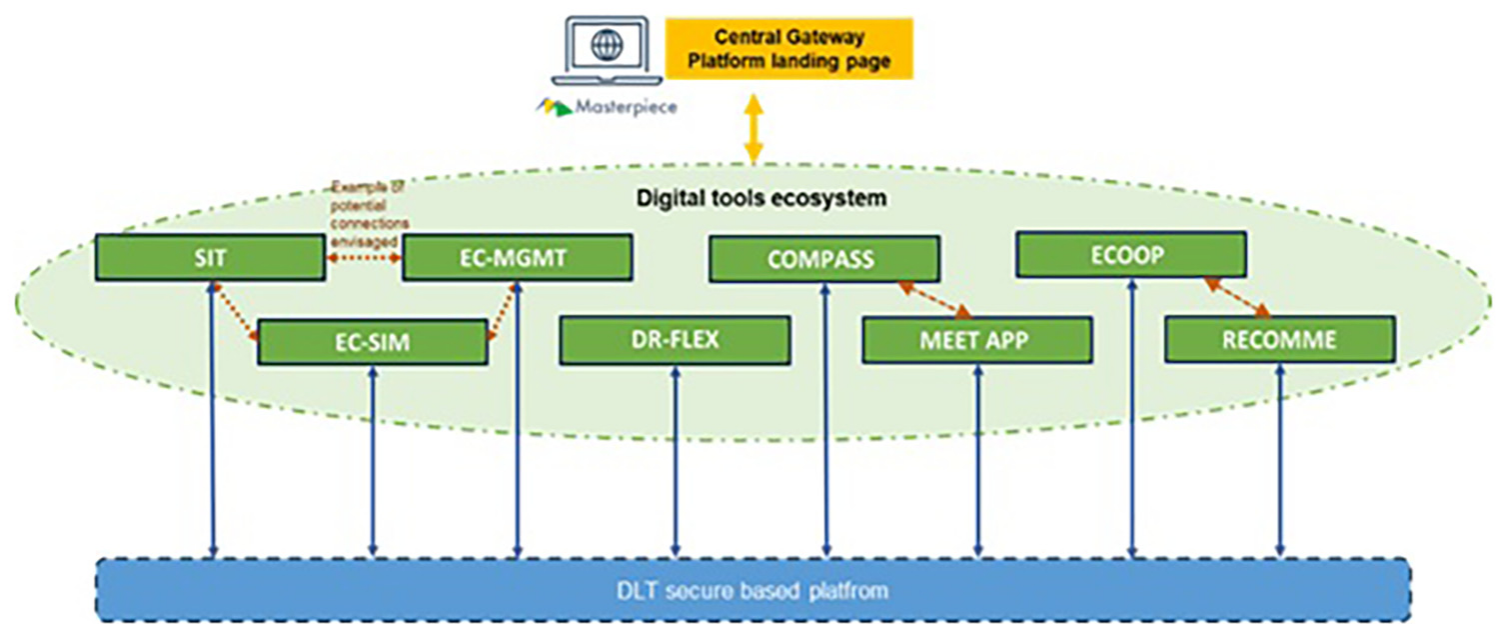
Scheme of the Masterpiece Concept.

The concept of the Masterpiece is illustrated in Figure 1. A platform is implemented where data (consumers/prosumers, etc.) from the pilot via APIs flow is exchanged with a digital tool ecosystem composed of a series of apps developed to foster the integration and realization of the renewable energy community. For Berchidda, Compass and Meet apps were adopted. The aim is to bring the local population closer to the active use of an energy community through events, workshops, and app adoption.

**Figure 2.**
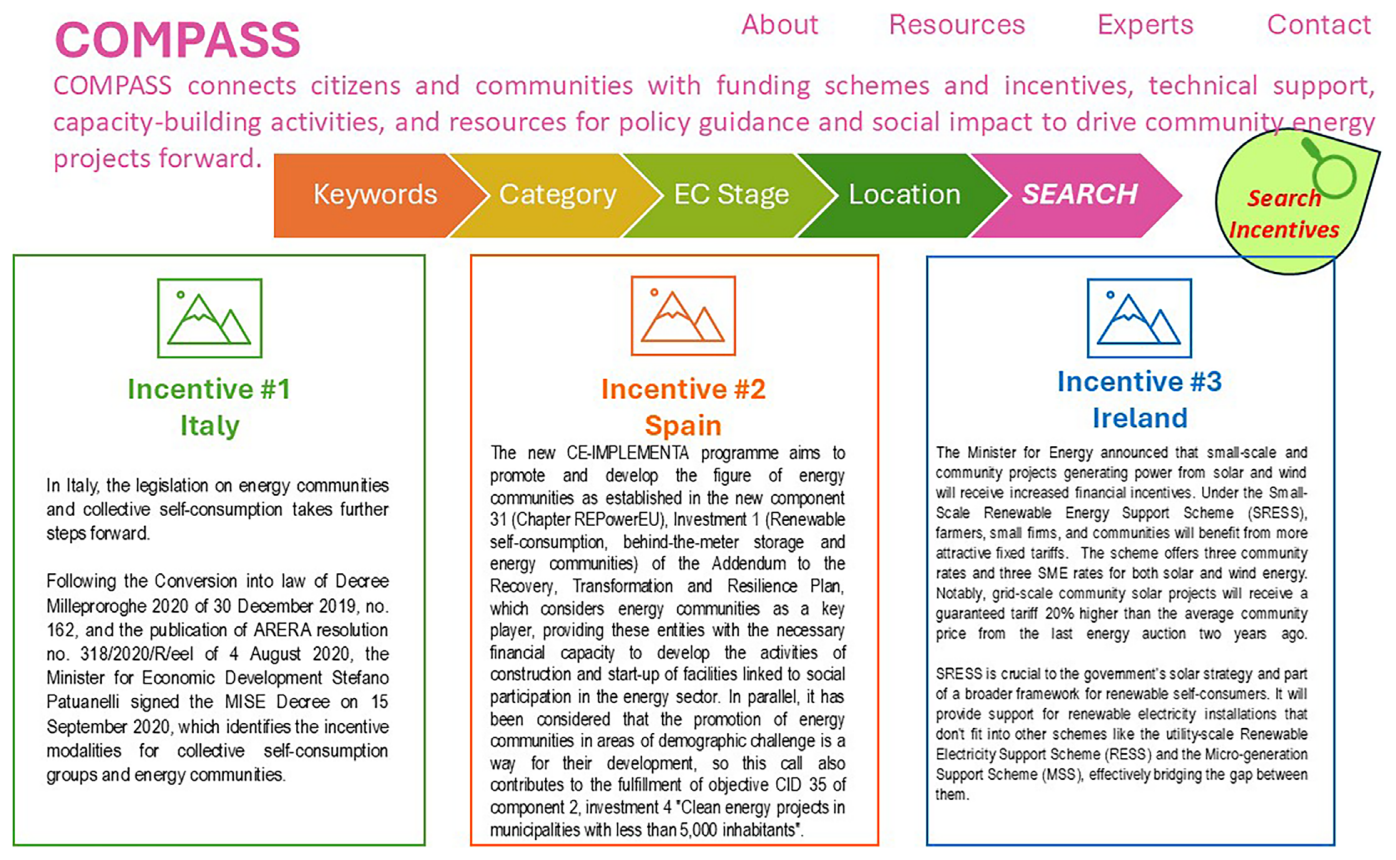
Example of Compass app.

An example of a compass app is shown in Figure 2. The Compass app connects the local community with a wide range of services that exist, but it is sometimes difficult for ordinary citizens to access. Masterpiece, by using a platform that communicates data interfacing with the apps, aims to put the local community at the center of the energy community so that it becomes the main actor, not just as a user, but as an active and responsible subject, to allow the REC itself to expand and prosper.

Among other things, Berchidda shows an interesting fact: According to the Italian National Statistics Office (ISTAT), women's literacy is double that of men, with women holding 30% more diplomas than men. For this reason, Masterpiece intended to target, with workshops and the use of apps, a female audience with a twofold purpose: to further integrate women into the use of and participation in RECs, carrying out a true empowerment activity, and to focus on women – the true economy at home – to have a successful REC.

### LOCALRES

LocalRES is a project geared towards the creation of renewable energy communities and focuses on the exploitation of their potential to promote the active involvement of citizens in the awareness of the importance of energy and its optimum management of residential properties. Within the project, a set of actions have been implemented to encourage sector coupling strategies and increase the penetration of renewable energies in the local context. These actions include both individual/building-level actions (e.g., photovoltaic plants, micro wind turbines, replacement of individual gas boilers with heat pumps) and community-level actions like the installation of vehicle to grid (V2G) chargers for electric vehicles and of storage assets to increase flexibility in local grids.

In the Berchidda community, planned actions aim to enhance energy efficiency, increase the use of renewable energy sources, and reduce carbon footprint. Konnex (KNX) technology and smart meters may play pivotal roles in realizing these objectives, providing the necessary tools for efficient energy management, user engagement, and the integration of renewable energy sources within the future digital management of energy consumption in the homes of energy community participants. LocalRES is a “demonstration-to-market” project that aims to create a planning tool and cost-effective multi-energy management solution, deeply assessing all the technical and non-technical aspects for the future replication of the LocalRES approach. For this purpose, demonstration and replication activities, which will actively involve technological providers, communities, and individuals, will play a crucial role.

The project forecasts the possible full implementation of a KNX system for a fully electrified apartment, in which there are several controllable electrical loads and a smart meter that allows signals to be sent to the central energy management control system, controlling equipment decisions. This is expanded to consider entire digitalized homes, illustrating the effect of changing the daily consumption profile by increasing self-consumption through the operation of heat pumps that match the production of photovoltaics, producing indirect thermal storage in residential buildings. Through this project, it was proven that devices, and therefore KNX home automation systems, integrated with Internet-of-Things (IoT) systems and new emerging technologies for smart buildings, can implement efficient and sustainable control and automation systems, leading to the integration of Smart Renewable Energy Communities, starting from digital residential buildings. Their synergy is essential for creating a robust infrastructure that supports collaborative energy practices, which can result in more efficient use of electricity, increasing the economic benefits related to incentive mechanisms for renewable energy communities. The use of KNX technologies allows for greater economic benefits related to the incentive mechanism for energy communities in place, which could be further increased through distributed storage systems among the premises of energy community users.

### SINNOGENES

SINNOGENES aims at designing and demonstrating the “Storage INNOvations (SINNO) energy toolkit,” a suite of innovative technologies and applications that render energy storage as the gamechanger for the acceleration of the successful energy transition and decarbonization towards an EU integrated and flexible energy system. Collaboration and sharing of experience across different countries is essential for learning the best paradigms for energy storage valorizations.

The key elements of the SINNOGENES approach are built upon and subsequently embraced:

- Innovative forms of energy storage: demonstration of innovative storage technologies through different applications in various energy carriers. Electrical, mechanical, electrochemical, and thermal storage are demonstrated in pilots in various configurations (e.g., behind-the-meter (BTM), front-of-the-meter (FTM), utility-scale, and off-grid). In particular, the following technologies will be developed: lithium-phosphate battery storage systems, V2G chargers for lithium-ion batteries in EVs, redox flow batteries, thermal battery storage, flywheels, ultracapacitors, power-to-gas (P2G) storage, Smart Heat storage, and digital replica of hydro-pumped storage;
- interoperability: scalability and interoperability of stand-alone and combined energy storage technologies, ensuring that they are compatible with different energy systems and architectures, from residential self-consumption and industrial microgrids (with electricity and thermal energy consumption) to distribution network management systems for distribution network applications;
- Various demand sectors and consumer segments: A diverse audience of consumers will be part of the project’s demonstration activities. Different segments of consumers, such as industrial, Local Energy Communities (LECs), services (offices, public buildings, etc.), residentials, and urban and maritime mobility, will offer a multidimensional landscape that strengthens the outcome of demonstration campaigns and thus creates an impact pathway for the adoption of innovative storage technologies across the EU;
- Technical requirements and market compliance for flexibility provision: The project focuses on harvesting the flexibility potential of assets existing in different levels of the energy system to provide flexibility services to operators through the utilization of innovative storage technologies (Peak-shaving, Fast Frequency Regulation, black start, energy arbitrage, congestion relief, dynamic regulation, etc.);
- Business model and decision-making investments for storage uptake: The project focuses on the creation of a framework that offers a holistic decision-making scheme for investments to promote the uptake of SINNOGENES energy storage technologies while boosting at the same time innovation and breakthroughs in energy storage systems.

SINNOGENES will support large-scale operations, with six demonstration campaigns across Europe in five dispersed countries (Portugal, Spain, Germany, Greece, and Switzerland). In particular, the demo site in Greece aims to valorize the hydro-pumped storage plant installed in Ikaria and evaluate the grid integration scenarios of the Samos and Ikaria islands. A feasibility study, empowered by the functionalities of the digital twin, will be conducted to explore the following aspects:

- The pumped-storage plant will provide frequency and non-frequency balancing services to both islands to alleviate the problems arising in the networks in case of a higher penetration rate of RES. This demonstration will leverage the capabilities of the digital replica of the hydro-storage plant to investigate the flexibility potential that the plant can provide to both Ikaria and Samos.
- Technical and economic assessment from the perspective of the hydro storage plant to study the deterioration/aging of the plant’s components owing to the increased utilization rate for the provision of services under different operational scenarios, as well as predictive maintenance capabilities.
- RES hosting capacity in both islands for different future demand scenarios and national decarbonization objectives.
- scenarios of the future grid integration of Samos with other North Aegean islands through high-voltage cables and further impacts of hydro-pumped storage valorization for island decarbonization.

These above-mentioned actions will lead to the following benefits:

- improved utilization of the existing hybrid storage plant in Ikaria;
- reduction in the energy generation’s environmental footprint in Samos Island;
- increase in the production of the other small hydro plants located at Ikaria;
- significant increase in the overall RES penetration rate in both Ikaria and Samos islands.

### SMHYLES

The SMHYLES project is a groundbreaking initiative with the aim of positioning European geographical islands as frontrunners in green energy transition. Launched in January 2024, the project focuses on the design and demonstration of hybrid energy storage systems (HESS) that are both sustainable and scalable. The primary goal is to develop safe energy storage solutions using low-critical raw materials (CRM) by leveraging innovative combinations of batteries and supercapacitors. These systems are designed for medium-to long-duration energy storage and the provision of multiple services, making them a crucial element in the renewable energy landscape. One of the key components of the project is its focus on real-world demonstration sites, each featuring different HESS configurations. The first demonstration will deploy a 400 kWh Aqueous HESS, consisting of an 8-hour vanadium redox flow battery (VRFB) and a 100 kW aqueous supercapacitor in a business park. This system provides extended energy storage, while supporting multiple grid services. The second demonstration will be conducted at an existing 2 MW renewable power plant, where it will be upgraded with a switchable RFB (redox flow battery) storage extension. This addition will improve weekly energy balancing and support future electric vehicle charging, while the inverter will be upgraded to form a virtual synchronous machine, enhancing the stability of the power system. The third and most island-relevant demonstration was planned for Graciosa Island in the Azores. This site hosts a 750 kWh salt-based HESS that integrates a smart ZEBRA battery with an aqueous supercapacitor, both with 250 kW nominal power. As an off-grid installation, this system is expected to significantly boost renewable energy integration and improve the grid stability. With these advancements, Graciosa can achieve renewable energy penetration rates close to 100%, further reducing its dependence on fossil fuels. A distinctive aspect of the SMHYLES project is the use of advanced digital tools for the optimization of HESS design and real-time energy management. An energy management system (EMS) integrates sophisticated algorithms that enable seamless operations, even in isolated island environments. For example, in Graciosa, the EMS enhances the existing Graciólica hybrid power plant, which already provides over 60% of the island’s energy from renewable sources. With the addition of HESS, the island’s renewable energy contribution could increase by an estimated 2.5% annually, reducing diesel consumption by approximately 350 MWh each year. In terms of technical advancements, SMHYLES hybrid energy storage systems can provide enhanced grid services. The HESS on Graciosa will not only help stabilize the island’s grid, but also improve energy density and reduce system losses by 15%. Through advanced energy dispatch strategies and the optimized use of renewable resources, the project aims to ensure a robust response to grid challenges, such as short-circuit events, while maintaining high levels of reliability and efficiency.

## Lessons learned and conclusions

This paper has presented part of the initiatives co-funded by the European Commission that are working on the energy transition and decarbonization of islands, leveraging the results of the workshop “EU Geographical Islands as Leaders of Green Energy Transition” held at the Sustainable Places 2024 conference.

The presented projects and their lessons learned, at different levels in line with the different levels of progress of the projects, some of which are completed and some of which are ongoing, have demonstrated that islands are characterized by features (barriers/opportunities) that make them ideal laboratories for the deployment of technical solutions and socio-economic approaches that can then be replicated on the mainland to support ambitious EU climate-related targets.

The main lessons learned through the review of the activities carried out in different R&D projects and their results include the following:

- The most suitable solution for the energy transition of a specific island strongly depends on the local features in terms of energy demand and supply potential and geographical and socio-economic features; for this reason, tailored energy planning plays a key role.
- islands are ideal laboratories for decarbonization solutions that can then be upscaled on the mainland, starting from renewable energy production coupled with different types of assets, like energy storage facilities, electric mobility on land and sea, desalination/water treatment, district heating/cooling, “power-to-X” solutions including the production of green hydrogen; moreover, all the technical solutions that are not specific for islands, like energy efficiency actions on buildings, industries and public assets, are applicable also to islands, generally with higher environmental, social and economic benefits;
- Energy communities have a high potential on islands since they leverage a strong sense of community that islanders have and allow increased security of supply, mitigation of energy poverty, and maximization of renewable energy self-consumption.
- Defining a standardized approach that addresses decarbonization in closed-grid systems can encourage public financial planning and private investment.
- Mapping and exploiting the RES potential is a key factor in reducing dependence on fossil fuels; a multiple-source approach can strengthen the penetration of RES into the power market, enhancing the autonomy of islands, and by combining smart grid solutions and innovative storage methods, the strong variation in the energy demand throughout the year can be tackled.
- Modern installations, equipped with smart meters, integrated in real-time energy management tools are emerging as yield boosters of energy efficiency, reducing losses, and optimizing energy use. The introduction of new storage technologies requires the development of advanced digital applications that can implement multi-factorial approaches that address new grid challenges.
- The development of modern digital applications and online platforms can facilitate cooperation among different actors in the power network and the interaction between separate energy communities that share common features and objectives, promoting pairing and combining innovative solutions that can be scalable, not exclusively to closed grid systems but also to mainland regions.
- The engagement of the local population in the energy transition, not only as users, but also as key participants of the projects is crucial to adequately develop energy communities; off-grid systems are characterized by a strong need for expertise and collaboration of each player in the network, and for this purpose, planning meetings and seminars on best practices in efficient energy management and sustainable decision-making helps build a relationship among the local population, public administration, and technical experts; moreover, the common effort for a self-reliance energy system will strengthen the sense of community on the island.

## Ethics and consent

No ethical approval or consent required.

## Data Availability

No data are associated with this article.
