# Supplementary material for: EU geographical islands as leaders of green energy transition
Source: Open Res Eur. 2025 Jul 4;4:258. Originally published 2024 Dec 4. [Version 2] doi: 10.12688/openreseurope.18856.2 (PMC12344401; doi:10.12688/openreseurope.18856.2)
Supplement: Peer review history

# Reviewer report: Reviewer response for version 2

**DOI:** 10.21956/openreseurope.22514.r56455

**Published:** 2025-09-11

**Authors:** Siamak Hosseinzadeh

**Affiliations:** 1 Sapienza University of Rome, Rome, Italy

Congratulation with this hard work. In my opinion, the paper has been improved significantly and could be considered for possible indexing.

Is the case presented with sufficient detail to be useful for teaching or other practitioners?

Partly

Is the work clearly and accurately presented and does it cite the current literature?

Partly

If applicable, is the statistical analysis and its interpretation appropriate?

Not applicable

Are all the source data underlying the results available to ensure full reproducibility?

Partly

Are the conclusions drawn adequately supported by the results?

Yes

Is the background of the case’s history and progression described in sufficient detail?

Partly

Reviewer Expertise:

NA

I confirm that I have read this submission and believe that I have an appropriate level of expertise to confirm that it is of an acceptable scientific standard.

# Reviewer report: Reviewer response for version 2

**DOI:** 10.21956/openreseurope.22514.r56792

**Published:** 2025-09-03

**Authors:** Romain Mauger

**Affiliations:** 1 Legal Research Unit, Iberian Centre for Research in Energy Storage (CIIAE), Cáceres, Spain

Summary of the article: This article presents 11 EU funded projects and initiatives about the energy transition on (mostly) EU islands. Based on these presentations, it gathers lessons learned to facilitate such transitions on islands first, and later on the mainland. The lessons learned cover a wide range of technologies, but also energy planning and social aspects.

 Notes:

 Overall, the necessary information is provided, although the quantify of references is quite on the low edge.

 Authors should provide links in footnote to all the mentioned projects’ websites or CORDIS pages. And when specific aspects of relevance are mentioned, they should be backed up by a source. For example, this should be done when the BRIDGE repository of best practices is mentioned at the end of p. 5; or on p. 6 when mentioning NESOI’s digital platform. And so on.

 All subsections presenting the projects should have pretty much the same structure, to provide a coherent story and allow for comparisons between projects (aims, locations, technical aspects, etc.). The REACT project’s text does not specify where the innovations are implemented at the start of the paragraphs. It drops names on p. 8 (Inis Mor, San Pietro, La Graciosa), without the reader having any idea about the countries where these places are.

 A quasi-similar situation happens with StepWise, where “Mediterranean islands” are mentioned but not their countries. Yet, especially with regards to local authorities’ powers, the country matters a lot.

 Another example of lack of coherence is that the funding programme as well as the starting year is indicated for project STEPWISE (p. 9), but not for the other projects. This should be streamlined and indicated for all projects.

 On p. 11, the statement “the true economy at home” is at the same time bold, unclear, unsourced and potentially conveying gendered clichés. I strongly suggest to delete it.

 The article reads well although there are some language mistakes in some occasions. A few are mentioned hereafter:

 p. 5: “In addition, the difficulties in public administration, whose authorities are often separated from the mentioned regions, and the lack of expertise regarding energy transition-specific planning results in a deceleration on the path to decarbonization and limiting energy self-sufficiency.” => the verb should be written “result”, without “s”.

 p. 8: The term “permits” or “authorizations” is usually used, rather than “permissions”.

 p. 10: the sentence starting with “At the same time, …” has an issue. Either delete the word “and” or another word is missing.

 It would be good to give another look at the whole article to correct any typo.

 .

 The text’s readability is sometimes impaired by very long paragraphs without space. This is for instance the case on pp. 8-9 in the IANOS project part. The text should be made more readable by giving space between paragraphs of between 10 and 20 lines approximately.

 The last sentence of the ISLET project on p. 10 is difficult to understand. Please revise writing.

 The scheme on p. 11 is of poor quality. Would be great if it could be replaced by a more readable one.

Is the case presented with sufficient detail to be useful for teaching or other practitioners?

Partly

Is the work clearly and accurately presented and does it cite the current literature?

Partly

If applicable, is the statistical analysis and its interpretation appropriate?

Not applicable

Are all the source data underlying the results available to ensure full reproducibility?

Partly

Are the conclusions drawn adequately supported by the results?

Yes

Is the background of the case’s history and progression described in sufficient detail?

Yes

Reviewer Expertise:

Energy law, Regulation of smart grid technologies, Regulation for islands' transition

I confirm that I have read this submission and believe that I have an appropriate level of expertise to confirm that it is of an acceptable scientific standard.

# Reviewer report: Reviewer response for version 2

**DOI:** 10.21956/openreseurope.22514.r56789

**Published:** 2025-08-12

**Authors:** GABRIEL WINTER

**Affiliations:** 1 Universidad de Las Palmas de Gran Canaria, Las Palmas de Gran Canaria, Canary Islands, Spain

This paper summarizes progress made by a large number of EU co-funded projects related to islands. Each of the selected projects contributing to the document focuses on the energy transition of European islands, mentioning different technologies such as renewable energy production systems, smart grids, energy storage systems, and local energy community schemes. The results of the analyzed projects highlight the need for energy planning adapted to geographical and socioeconomic particularities. A set of lessons learned is outlined regarding the technologies with the greatest potential to be evaluated for implementation on islands and useful for replication on the European continent.

 The introduction is quite precise, well-written, and very clear.

 I have minor suggestions, but the manuscript can be approved.

 Suggestions:

 It is recommended to include somewhat more thorough review of the literature, where the novelty of the work presented in this article is better reinforced, considering especially its complementarity and points of interest in comparison to even recent articles.

 - to include the temporal data of the start year and end year of each of the cited projects.

 - to complete several more projects with high replicability ,  among the 15 best practices for replication highlighted from the 54 supported projects addressed in NESOI (1 Oct 2019 - Feb 2024), and objective information extracted from the Guidebook available online: **https://managenergy.ec.europa.eu/document/download/05ff7571-c198-41cd-8c31-ed85fc6743aa_en?filename=guidebook%20islands.pdf** Therefore I recommend to include the following two projects related to incorporating Green Hydrogen:

**- **The Z-175 Green Orkney Hydrogen Market Expansion project, which has been assessed as having a high replicability and exploitation potential, RRL = 2.8/3. *“ The project is based on proven hydrogen transport and storage technologies, the solution considers various scenarios, including distance, route and hydrogen volume transported. The project concept can be applied to any island by adapting the technological choices to the local context”*

*- The GHEKO Project. The replicability potential of the GHEKO project is indeed high (RRL=2.6/3), especially islands with a Port that which has a certain level of activity, even with buckering activities. “The generation of hydrogen is facilitated by taking advantage of the excess electricity produced by local wind farms, which currently face a significant limitation. The attractiveness of hydrogen, especially in utilizing the energy from already installed wind facilities, has generated great interest among local communities in the Non-Interconnected Islands (INI). Hydrogen, which emerges as a versatile resource for these communities , serves as a versatile solution, promoting efficient sector coupling. “*

 -Also maybe is interesting to include  The Z-254 POSIDON project that has been assessed as having a high replicability and operational potential, with a score of 2.6/3 *.”  The project involves setting up four energy communities using on solar PV power and shared sustainable mobility”*

 - Also to add the ISLANDER Project *ISLANDER *( Oct 2020 – Sep 2025)**** ***,*** *developed on the islands of Borum, Germany (ref:* *https://clean-energy-islands.ec.europa.eu/news/islander-start-installation-phase-island-borkum* *)* because of the starting with the Pilot island of Borkum, the project aims at replicating these solutions to 4 follower islands: Lefkada and Skopelos in Greece, Orkney in the UK and Cres in Croatia, and to other EU islands

 and it is interesting to mention the hybrid project GORONA DEL VIENTO (https://www.goronadelviento.es), an installation inaugurated in 2015, which combines wind turbines (11.5 MW) with hydraulic pumping, a closed energy storage system, with coverage of consumption during hours with little wind or level of water to produce enough energy to meet demand. The system has allowed for operation without diesel for many cumulative hours, supplying up to 50% of annual electricity demand with renewable sources and reducing thousands of tons of CO2, becoming a global model of energy self-sufficiency in isolated territories.

Is the case presented with sufficient detail to be useful for teaching or other practitioners?

Yes

Is the work clearly and accurately presented and does it cite the current literature?

Partly

If applicable, is the statistical analysis and its interpretation appropriate?

Not applicable

Are all the source data underlying the results available to ensure full reproducibility?

Yes

Are the conclusions drawn adequately supported by the results?

Yes

Is the background of the case’s history and progression described in sufficient detail?

Yes

Reviewer Expertise:

INTELLIGENT SYSTEMS AND APPLICATIONS IN ENGINEERING, SIMULATION AND OPTIMIZATION OF ENERGY SYSTEMS

I confirm that I have read this submission and believe that I have an appropriate level of expertise to confirm that it is of an acceptable scientific standard.

# Reviewer report: Reviewer response for version 1

**DOI:** 10.21956/openreseurope.20406.r48073

**Published:** 2024-12-19

**Authors:** Avraam Kartalidis

**Affiliations:** 1 Centre for Research and Technology Hellas (CERTH), Thermi, Thessaloniki, Greece

This paper successfully summarizes the outcomes/progress of a significant number of EU-funded projects regarding the geographical islands. The introduction is very well written and gives a very clear message. 

 One of the main arguments is that the current energy cost is very high in the non-interconnected islands, thus the application of renewable energy or other innovative solutions is feasible and cost-effective. This is true but it is not a significant driving force for the locals to embrace new technologies, as the actual price that the locals pay for energy is the same as in the mainland or other systems. The mainland residents/consumers are paying for the island communities, strictly speaking.

 I have minor suggestions but the manuscript can be approved. Suggestions for the published article.

 This paper can be a very important source of information for locals in order to start replication activities and to search for results and practices from the projects. Thus a short comparison sheet among the various projects is nice to have for reference purposes.

 Two important factors that can be mentioned

1. The vast difference between the islands itself. Not a great number of island participates in these projects. That situation extends the gap among islands that might be very close geographically
2. The early adopter experience. It is important for the projects to succeed as a bad experience will discourage the local communities from participating and will provide a stagnation to similar technologies development/application, even if they become mature.

Comments- fix bullets (separators etc)

 Not clear what is going on with STEPWISE’s islands.

 The project addresses municipalities in four use cases: Bulgaria, Spain, Cyprus, and the Mediterranean islands.

 Currently, the case involves three pilots: Cres, Crete, and Malta. Along with other islands, pilots can become adopters and develop their own energy plans.

 Not clear the bullet in ISLET

 the water-energy nexus addressed simultaneously is a combination of island projects;

Is the case presented with sufficient detail to be useful for teaching or other practitioners?

Yes

Is the work clearly and accurately presented and does it cite the current literature?

Yes

If applicable, is the statistical analysis and its interpretation appropriate?

Not applicable

Are all the source data underlying the results available to ensure full reproducibility?

Not applicable

Are the conclusions drawn adequately supported by the results?

Yes

Is the background of the case’s history and progression described in sufficient detail?

Yes

Reviewer Expertise:

Renewable energy systems. Water Energy Nexus. Technoeconomic Evaluation.

I confirm that I have read this submission and believe that I have an appropriate level of expertise to confirm that it is of an acceptable scientific standard.

# Reviewer report: Reviewer response for version 1

**DOI:** 10.21956/openreseurope.20406.r48069

**Published:** 2024-12-13

**Authors:** Siamak Hosseinzadeh

**Affiliations:** 1 Sapienza University of Rome, Rome, Italy

1. To strengthen the impact and significance of your abstract, consider expanding on the claim that European islands can drive the EU's decarbonization strategy. Additionally, explore the future role of islands in green energy generation.
2. To enhance the clarity and accessibility of your abstract, please consider providing the full form of any abbreviations or acronyms used, followed by the abbreviated form in parentheses.
3. To enhance the readability and clarity of your paper, consider adding a table of nomenclature or a list of abbreviations and symbols.
4. The novelty of the work must be clearly addressed and discussed, considering recent articles weak points; compare your research with existing research findings; and highlight novelty. (Compare your work with existing research findings and highlight novelty.)
5. To strengthen the claim that lands have significant potential for renewable energy sources, consider providing more specific examples and citations to support this assertion.
6. To gain a comprehensive understanding of the RE-EMPOWERED project, it would be beneficial to investigate whether solar and PV-based solutions were considered or implemented as part of the project's objectives. This is particularly important because solar-based solutions can be extended to other projects, particularly those focused on renewable energy and sustainable development.
7. To enhance the clarity and comparability of the different solutions or projects discussed in your paper, consider creating a table that summarizes their key features and performance metrics.
8. To enhance the visual quality of Figure 2, consider using a higher resolution image format. This will result in a sharper image with clearer details, making it easier to interpret the information presented.
9. To strengthen the foundation of your research and highlight its unique contributions, it is recommended to include a comprehensive literature review.

Is the case presented with sufficient detail to be useful for teaching or other practitioners?

Partly

Is the work clearly and accurately presented and does it cite the current literature?

Partly

If applicable, is the statistical analysis and its interpretation appropriate?

Not applicable

Are all the source data underlying the results available to ensure full reproducibility?

Partly

Are the conclusions drawn adequately supported by the results?

Yes

Is the background of the case’s history and progression described in sufficient detail?

Partly

Reviewer Expertise:

N/A

I confirm that I have read this submission and believe that I have an appropriate level of expertise to confirm that it is of an acceptable scientific standard, however I have significant reservations, as outlined above.
